# Prevalence of soil-transmitted helminths and associated risk factors among primary school children in Kandahar, Afghanistan: A cross-sectional analytical study

**DOI:** 10.1371/journal.pntd.0011614

**Published:** 2023-09-11

**Authors:** Bilal Ahmad Rahimi, Najeebullah Rafiqi, Zarghoon Tareen, Khalil Ahmad Kakar, Mohammad Hashim Wafa, Muhammad Haroon Stanikzai, Mohammad Asim Beg, Abdul Khaliq Dost, Walter R. Taylor

**Affiliations:** 1 Department of Pediatrics, Faculty of Medicine, Kandahar University, Kandahar, Afghanistan; 2 Department of Pediatrics, Faculty of Medicine, Malalay Institute of Higher Education, Kandahar, Afghanistan; 3 Department of Surgery, Faculty of Medicine, Kandahar University, Kandahar, Afghanistan; 4 Department of Public Health, Faculty of Medicine, Malalay Institute of Higher Education, Kandahar, Afghanistan; 5 Department of Psychiatry, Faculty of Medicine, Kandahar University, Kandahar, Afghanistan; 6 Department of Public Health, Faculty of Medicine, Kandahar University, Kandahar, Afghanistan; 7 Department of Pathology and Laboratory Medicine, The Aga Khan University Hospital, Karachi, Pakistan; 8 Mahidol Oxford Tropical Medicine Clinical Research Unit (MORU), Mahidol University, Bangkok, Thailand; Swiss Tropical and Public Health Institute, SWITZERLAND

## Abstract

**Background:**

Soil-transmitted helminth (STH) infections are global health problem, especially in low-income countries. Main objectives of this study were to estimate the prevalence and intensity of STH and its risk factors among school children in Kandahar city of Afghanistan.

**Methodology/principal findings:**

This was a school-based cross-sectional analytical study, with data collected during eight-month-period (May–December, 2022) from 6- and 12-years old school children in Kandahar city, Afghanistan. All the stool samples were examined by saline wet mount method and Kato–Katz technique. Data were analyzed by using descriptive statistics, Chi square test, and multivariate logistic regression.

A total of 1275 children from eight schools of Kandahar city were included in this study. Mean age of these children was 8.3 years with 53.3% boys. The overall prevalence of any intestinal parasitic infection was 68.4%. The overall prevalence of STH infection was 39.1%, with *Ascaris lumbricoides* (29.4%) as the most prevalent STH species. Mean intensity of overall STH infection was 97.8. Multivariate logistic regression revealed playing barefoot (AOR 1.6, 95% CI 1.1–2.2), not washing hands after defecating and before eating (AOR 1.3, 95% CI 1.0–1.7), having untrimmed nails (AOR 1.4, 95% CI 1.1–1.8), and belonging to poor families (AOR 1.3, 95% CI 1.0–1.7) as the risk factors associated with the predisposition of school children for getting STH in Kandahar city of Afghanistan.

**Conclusions/significance:**

There is high prevalence of STH among school children of Kandahar city in Afghanistan. Most of the risk factors are related to poverty, decreased sanitation, and improper hygiene. Improvement of socioeconomic status, sanitation, and health education to promote public awareness about health and hygiene together with periodic mass deworming programs are better strategies for the control of STH infections in Afghanistan.

## Author summary

Soil-transmitted helminths (STH) are a group of intestinal parasites which globally infect more than 1.5 billion people, primarily in low- and middle-income countries. Approximately 260 million preschool-age and 654 million school-age children live in areas where the transmission of STH is very high. The risk of parasitic diseases is estimated to be very high in Afghanistan, but studies are rarely done on STH in Afghanistan. So, this study was conducted with main goals to estimate the prevalence and intensity of STH and its associated factors among primary school children in Kandahar city of Afghanistan. This study found that prevalence of STH is high among school children. Most of the factors associated with increased STH infections in Kandahar city school children are related to poverty, decreased sanitation, and improper hygiene. Improvement of socioeconomic status, sanitation, and health education to promote public awareness about health and hygiene together with periodic mass deworming programs are better strategies for the control of STH infections in Afghanistan.

## Introduction

Soil-transmitted helminths (STH) belong to a group of neglected tropical diseases, which occur primarily in low- and middle-income countries across tropical and subtropical regions, and disproportionately affect low-income communities [1]. Globally, World Health Organization (WHO) estimates more than 1.5 billion (24% of the world population) people are infected with STHs in Africa, Asia, and Latin America [1–3]. Over 260 million preschool-age and 654 million school-age children live in areas where STH transmission is very high [1].

Globally, the most prevalent STH is *Ascaris lumbricoides* (*A*. *lumbricoides*) (infecting approximately 1.2 billion people), followed by *Trichuris trichiura* (*T*. *trichiura*) (infecting nearly 795 million people) and hookworm (*Ancylostoma duodenale* and *Necator americanus*) which infects approximately 740 million people [4,5]. School age children are at high risk of being infected with STH. This could be due to the reason that these children are more exposed to contaminated soil when they play, walk barefoot, eat soil, and do not practice good personal hygiene [6]. Individuals with moderate-to-heavy intensity STH infections experience adverse health outcomes including diarrhea, abdominal pain, anemia, and impaired cognitive and physical development in children [5,7–11]. Deworming campaigns in different countries of the world have shown to improve nutritional status, cognition, and school performance in school-age children [12–14].

Afghanistan, for the last four decades, has been suffering from military and civil conflict. This, combined with natural disasters, has extremely weakened economic development [15]. The prevalence of STH is mostly unknown in Afghanistan. Diagnosis is mostly made on clinical basis without any laboratory confirmation. The advent of Taliban government, decrease in international humanitarian aid, insecurity, and shortage of medical staff at all levels of the healthcare system hinders the implementation of epidemiological surveillance [16]. Healthcare system in Afghanistan is mostly dependent of international humanitarian aid [17]. The risk of parasitic diseases is estimated to be very high in Afghanistan [18].

Unfortunately, to our knowledge, only two studies have been conducted in Afghanistan to find the prevalence of STH among primary school children [19,20]. In 2003, World Health Organization (WHO) conducted a school-based survey with the help of Afghan ministry of public health and Afghan ministry of education. They collected fecal samples from 1,001 school children in four provinces of Afghanistan (Kabul, Kandahar, Nangarhar, and Farah) for soil-transmitted helminths (STH) [19]. In 2017, a follow-up survey was conducted among school children aged 8–10 years to provide an update on STH epidemiology in Kabul, Balkh, Herat, Nangarhar, and Kandahar provinces of Afghanistan [20]. In 2020, a community-based study among 1426 children was conducted to estimate the prevalence and associated factors of STH among children in Daman district of Kandahar province in Afghanistan. In this study, the overall prevalence of STH infection was 22.7%. Main risk factors associated with the predisposition of rural children for getting STH were not washing hands after defecating/before eating, living in mud house, walking barefoot, living in overcrowded house, and practicing open defecation [21]. In another hospital-based study in Afghanistan, 548 fecal samples were collected from the patients (both children and adults) with internal complaints who were admitted in two hospitals of Ghazni and Parwan provinces. More than one-third of these patients had intestinal helminths [16]. Main objectives of this school-based study were to estimate the prevalence and intensity of STH and its risk factors among school children in Kandahar city of Afghanistan.

## Methods

### Ethics statement

Written informed consents were taken from parents or guardians of all the participants prior to the study. Also, assent forms were given to the children for the participation. Information of the participants will not be disclosed. Ethical approval was taken from Kandahar University Ethics Committee (code number KDRU-EC-2022.19). For data collection, only children’s initials were used. Prior to entering into the computer for analysis, the collected data was coded and de-identified. Also, to minimize the errors, data was double entered.

### Study design and study area

This was a school-based cross-sectional analytical study, conducted during eight-month-period (May–December, 2022) in Kandahar. Kandahar is a city, located in the south-west of Afghanistan. This city is located at an elevation of 1,010 meters. Kandahar is Afghanistan’s second largest city after Kabul, with a population of about 614,118 people. All the schools of Kandahar city were selected for randomization using lottery method. After randomization, eight schools (four boys’ schools and four girls’ schools) were selected for the study. The four boys’ school were Ahmad Shah Baba school, Mirwais Neka school, Zahir Shahi school, and Temor Shahi school. The four girls’ schools were Zarghona Ana school, Malalai school, Nazo Ana school, and Aino Ana school.

### Study population and sample size calculation

Our source population was comprised of primary school children (both boys and girls) of class one to class five, with ages between 6 and 12 years. All those children were excluded from the study who received any anti-helminthic treatment in the previous three months before the commencement of the study, having chronic diseases, not able to provide stool samples, or their parents/guardians refused to participate in the study. If more than one children are coming from the same household, the statistics can be over represented in the household with many primary school age children. To avoid this issue, only one child per house was enrolled in this study.

The sample size and power calculations were performed in Epi Info version 7.2 (CDC, Atlanta, Georgia, USA). A 20% non-response rate was added. Our sample size was 1385 children. Among these children, parents/guardians of 32 (2.3%) children refused to take part in the study, 70 (5.1%) failed to submit their fecal samples, six (0.4%) had history of receiving anti-helminthic treatment in the last three months, while two (0.1%) had history of chronic disease (thalassemia). So, data was collected from 1275 children.

The total population of Kandahar city is 614,118 with primary school age population of 121,000 children. During 2022, there were a total of 17,950 (14.8% of primary school age population) primary school children with 11844 (66%) boys and 6,106 (34.0%) girls [22]. So, our sample size constitutes 7.1% of school population while 1.1% of all primary school-age children of Kandahar city.

### Sample collection and laboratory procedures

From all schools, children were selected using lottery method of randomization. A questionnaire was utilized in two local languages (Pashto and Dari) with questions regarding general characteristics, economic status, general sanitation and environmental conditions, and laboratory examination. Data were collected/recorded on paper forms by experienced and trained investigators. Prior to data collection, short briefings were given, during which the objectives and methods of the study were clearly informed to the children. The case record form and other materials were pretested before the actual data collection. Persons responsible for data collection were well trained on how to conduct the interview with children and their parents or care-takers and how to collect the stool samples. For stool sample collection, the children who agreed to participate in the study were provided with clean pre-labeled capped plastic container for stool collection along with instruction on correct placement of the stool into the containers. All the children were instructed to collect 100 mg of the stool samples. In the laboratory, saline wet mount method and Kato–Katz technique were used by expert laboratory technicians. Saline wet mount method was used for protozoa identification while Kato-Katz technique was used to detect and find the intensity of intestinal helminths. Kato-Katz thick smears were examined within one hour of its preparation to avoid over clearing of Hookworm eggs. The total number of eggs were expressed as eggs per gram (EPG) of stool. EPG was calculated to classify the infection intensity as light, moderate, and heavy infection. The severity of STHs infection is defined as light, moderate, and heavy intensity of infections, respectively, as follows *A*. *lumbricoides*: 1 to 4999 EPG, 5000 to 49999 EPG, and ≥50000 EPG; *T*. *trichiura*: 1 to 999 EPG, 1000 to 9999 EPG, and ≥10000 EPG; hookworm: 1 to 1999 EPG, 2000 to 3999 EPG, and ≥4000 EPG [23]. For quality control, 10% of the stool samples were randomly selected and examined by another experienced laboratory technician who was blinded for the previous test result.

### Data analysis

The data were entered into Microsoft Excel, cleaned, and imported to Statistical Package for the Social Sciences (SPSS) version 22 (Chicago, IL, USA) for statistical analysis. Descriptive analysis including frequency, mean, standard deviation (SD), and range was used to summarize demographic characteristics. Frequency and percentage were used to summarize categorical variables. Chi-square test (using crude odds ratio [COR]) was performed to assess the binary association between various categorical variables. All variables that were statistically significant in univariate analyses were assessed for independence in a multivariate logistic regression (using adjusted odds ratio [AOR]) to determine the factors associated with the predisposition of rural children for getting STH. A *P*-value of <0.05 was statistically significant.

## Results

### Socio-demographic, economic characteristics, and hygiene conditions

Among 1385 children, data was collected from 1275 children. So, our response rate was 92.1%. Among 679 (53.3%) boys, 190 (14.9%) were from Temor Shahi school, 176 (13.8%) from Mirwais Neka school, 161 (12.6%) from Ahmad Shah Baba school, and 152 (11.9%) from Zahir Shahi school. From 596 (46.7%) girls, 152 (11.9%) were from Malalai school, 150 (11.8) from Nazo Ana school, 148 (11.6%) from Aino Ana school, and 146 (11.5%) from Zarghona Ana school. Mean (SD) age of these children was 8.3 (1.8) years with 903/1275 (70.8%) belonging to poor families (daily income <2 USD per day) (Table 1).

**Table 1:** Socio-demographic and other characteristics of the study participasnts.

Variable	Total, n (%) (n = 1275)	Boys, n (%) (n = 679)	Girls, n (%) (n = 596)	*P*-value
STH present in stool				
Yes	499 (39.1)	270 (39.8)	229 (38.4)	0.624
No	776 (60.9)	409 (60.2)	367 (61.6)	
Family daily income per person (in Afghanis)				
≥180 (≥2 USD)	372 (29.2)	210 (30.9)	162 (27.2)	0.142
<180 (<2 USD)	903 (70.8)	469 (69.1)	434 (72.8)	
Family size				
<5 people	297 (23.3)	151 (22.2)	146 (24.5)	0.341
≥5 people	978 (76.7)	528 (77.8)	450 (75.5)	
Mother’s education				
Literate	149 (11.7)	82 (12.1)	67 (11.2)	0.643
Illiterate	1126 (88.3)	597 (87.9)	529 (88.8)	
Father’s education				
Literate	300 (23.5)	137 (20.2)	163 (27.3)	0.003
Illiterate	975 (76.5)	542 (79.8)	433 (72.7)	
House construction				
Concrete	313 (24.5)	198 (29.2)	115 (19.3)	<0.001
Mud	962 (75.5)	481 (70.8)	481 (80.7)	
Source of drinking water				
Safe	1010 (79.2)	509 (75.0)	501 (84.1)	<0.001
Unsafe	265 (20.8)	170 (25.0)	95 (15.9)	
Toilet in school				
Absent or not functional	565 (44.3)	179 (26.4)	386 (64.8)	<0.001
Present and functional	710 (55.7)	500 (73.6)	210 (35.2)	
Washing hands after defecating/before eating				
Yes	419 (32.9)	233 (34.3)	186 (31.2)	0.239
No	856 (67.1)	446 (65.7)	410 (68.8)	
Walking barefoot				
Yes	180 (14.1)	99 (14.6)	81 (13.6)	0.613
No	1095 (85.9)	580 (85.4)	515 (86.4)	
Finger nail status				
Untrimmed	866 (67.9)	467 (68.8)	399 (66.9)	0.485
Trimmed	409 (32.1)	212 (31.2)	197 (33.1)	
Habit of nail biting				
Yes	115 (9.0)	60 (8.8)	55 (9.2)	0.808
No	1160 (91.0)	619 (91.2)	541 (90.8)	
Consumption of raw vegetables				
Yes	776 (60.9)	266 (39.2)	510 (85.6)	<0.001
No	499 (39.1)	413 (60.8)	86 (14.4)	
Habit of eating soil				
Yes	70 (5.5)	50 (7.4)	20 (3.4)	0.002
No	1205 (94.5)	629 (92.6)	576 (96.6)	
Domestic animals present at home				
Yes	315 (24.7)	219 (32.3)	96 (16.1)	<0.001
No	960 (75.3)	460 (67.7)	500 (83.9)	

N, Number; STH, Soil-transmitted helminth; USD, United States Dollar.

### Prevalence of soil-transmitted helminths

**Fig 1:**
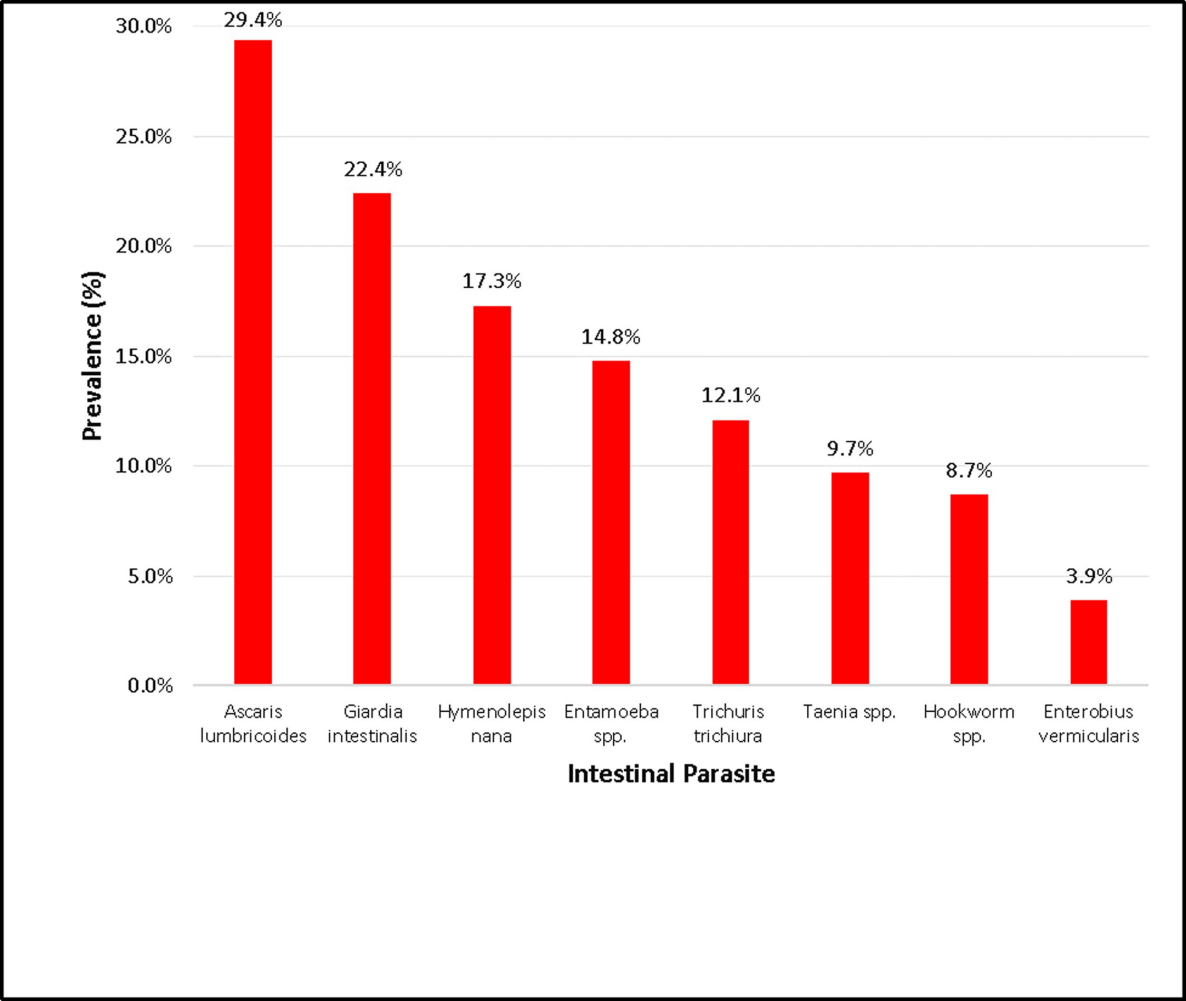
Prevalence of intestinal parasites among school children in Kandahar city.

The overall prevalence of STH infection was 39.1% (499/1275 children). *A*. *lumbricoides* (29.4%, 375/1275) was the most prevalent STH species, followed by *T*. *trichiura* (12.1%, 154/1275) and hookworm (8.7%, 111/1275). Prevalence of intestinal protozoa infection was 31.7% (404/1275) while prevalence of overall any intestinal parasitic infection was 68.4% (872/1275). *G*. *intestinalis* (*Giardia intestinalis*) was the most prevalent intestinal protozoa with a prevalence of 22.4%. Among the STH infected patients, single infection, double infection, and triple infections were present in 343/1275 (26.9%), 151/1275 (11.8%), and 5/1275 (0.4%) of the children, respectively. Among other intestinal parasites, *Hymenolepis nana* was the most prevalent (221/1275 [17.3%]) (Table 2 and Fig 1).

**Table 2:** Species of intestinal parasitic infection among primary school children in Kandahar city.

Intestinal parasitic infection	Number (n = 1275)	Prevalence (%)
Overall any intestinal parasitic infection	872	68.4
Monoparasitism	474	37.2
Polyparasitism	398	31.2
Overall any STH infection	499	39.1
Single STH infection	343	26.9
Double STH infection	151	11.8
Triple STH infection	5	0.4
Overall any intestinal protozoa infection	404	31.7
STH		
*Ascaris lumbricoides*	375	29.4
*Trichuris trichiura*	154	12.1
Hookworm spp.	111	8.7
Protozoa		
*Giardia intestinalis*	286	22.4
Entamoeba spp.	189	14.8
Other intestinal parasites		
*Hymenolepis nana*	221	17.3
Taenia spp.	124	9.7
*Enterobius vermicularis*	50	3.9

n, Number; STH, Soil-transmitted helminth.

### The intensity of soil-transmitted helminths

The intensity of STHs infection was categorized based on the WHO classification thresholds using Kato-Katz thick smear method of parasites egg quantification expressed in eggs per gram (EPG) of stool. Mean intensity of overall STH infection was 97.8. The mean intensity of *A*. *lumbricoides*, *T*. *trichiura*, and hookworm spp. infection in our study was 105.4, 92.8, and 95.5, respectively. Light intensity of *A*. *lumbricoides*, *T*. *trichiura*, and hookworm spp. was present in 90.4% (339/375), 97.4% (150/154), and 95.5% (106/111) of the school children, respectively. Heavy intensity of STH was not observed in any of the children (Table 3).

**Table 3:** Infection intensity of STHs among primary school children in Kandahar city.

	Frequency, n (%)	EPG, mean (range)
Overall STH infection	n = 499	97.8 (2–13150)
*Ascaris lumbricoides*		
Overall	n = 375	105.4 (5–13150)
Light intensity	339 (90.4)	103.6 (5–4370)
Moderate intensity	36 (9.6)	6380 (5600–13150)
Heavy intensity	0	0
*Trichuris trichiura*		
Overall	n = 154	92.8 (3–1650)
Light intensity	150 (97.4)	90.3 (3–780)
Moderate intensity	4 (2.6)	1375 (1200–1650)
Heavy intensity	0	0
Hookworm spp.		
Overall	n = 111	95.5 (2–2800)
Light intensity	106 (95.5)	92.4 (2–1680)
Moderate intensity	5 (4.5)	2250 (2180–2800)
Heavy intensity	0	0

EPG, Eggs per gram; spp, Species.

### Risk factors of soil-transmitted helminths

In Chi-square test, statistically significant variables responsible for increased STH infection were children playing barefoot (COR 1.7, 95% CI [confidence interval] 1.2–2.3, and *p*-value 0.001), not washing hands after defecation and before eating (COR 1.5, 95% CI 1.1–1.9, and *p*-value 0.002), having untrimmed finger nails (COR 1.5, 95% CI 1.2–1.9, and *p*-value 0.001), habit of nail biting (COR 1.5, 95% CI 1.0–2.2, and *p*-value 0.045), and belonging to poor families (COR 1.4, 95% CI 1.1–1.8, and *p*-value 0.009) (Table 4).

**Table 4:** Univariate analyses and logistic regression of risk factors associated with increased STH in primary school children.

Variable	Total, n (%) (n = 1275)	STH infection present		COR (95% CI)	*P*-value	AOR (95% CI)	*P*-value
		Yes, n (%) (n = 499)	No, n (%) (n = 776)				
Gender							
Male	679 (53.3)	270 (39.8)	409 (60.2)	0.9 (0.8–1.2)	0.624		
Female	596 (46.7)	229 (38.4)	367 (61.6)	1			
Mother’s education							
Literate	149 (11.7)	62 (41.6)	87 (58.4)	1	0.510		
Illiterate	1126 (88.3)	437 (38.8)	689 (61.2)	1.1 (0.8–1.6)			
Father’s education							
Literate	300 (23.5)	122 (40.7)	178 (59.3)	1	0.535		
Illiterate	975 (76.5)	377 (38.7)	598 (61.3)	1.1 (0.8–1.4)			
Playing barefoot						1.6 (1.1–2.2)	
Yes (risky)	180 (14.1)	90 (50.0)	90 (50.0)	1.7 (1.2–2.3)	0.001		0.006
No	1095 (85.9)	409 (37.4)	686 (62.6)	1			
House construction							
Concrete	313 (24.5)	113 (36.1)	200 (63.9)	1	0.205		
Mud	962 (75.5)	386 (40.1)	576 (59.9)	0.8 (0.6–1.1)			
Source of drinking water							
Safe	1010 (79.2)	391 (38.7)	619 (61.3)	1	0.544		
Unsafe	265 (20.8)	108 (40.8)	157 (59.2)	0.9 (0.7–1.2)			
Family daily income per person (in Afghanis)							
≥180 (≥2 USD)	372 (29.2)	125 (33.6)	247 (66.4)	1	0.009	1	0.038
<180 (<2 USD)	903 (70.8)	374 (41.4)	529 (58.6)	1.4 (1.1–1.8)		1.3 (1.0–1.7)	
Family size							
<5 people	297 (23.3)	119 (40.1)	178 (59.9)	1	0.708		
≥5 people	978 (76.7)	380 (38.9)	598 (61.1)	1.1 (0.8–1.4)			
Washing hands after defecating/before eating							
Yes	419 (32.9)	139 (33.2)	280 (66.8)	1	0.002	1	0.043
No (risky)	856 (67.1)	360 (42.1)	496 (57.9)	1.5 (1.1–1.9)		1.3 (1.0–1.7)	
Finger nails status							
Untrimmed (risky)	866 (67.9)	365 (42.1)	501 (57.9)	1.5 (1.2–1.9)	0.001	1.4 (1.1–1.8)	0.010
Trimmed	409 (32.1)	134 (32.8)	275 (67.2)	1		1	
Habit of nail biting							
Yes	115 (9.0)	55 (47.8)	60 (52.2)	1.5 (1.0–2.2)	0.045	1.0 (0.6–1.6)	0.944
No	1160 (91.0)	444 (38.3)	716 (61.7)	1			
Consumption of raw vegetables							
Yes	776 (60.9)	292 (37.6)	484 (62.4)	1	0.169		
No	499 (39.1)	207 (41.5)	292 (58.5)	0.9 (0.7–1.1)			
Habit of eating soil							
Yes	70 (5.5)	29 (41.4)	41 (58.6)	1	0.686		
No	1205 (94.5)	470 (39.0)	735 (61.0)	1.1 (0.7–1.8)			
Domestic animals present at home							
Yes	315 (24.7)	130 (41.3)	185 (58.7)	0.9 (0.7–1.2)	0.371		
No	960 (75.3)	369 (38.4)	591 (61.6)	1			
Toilet in school							
Absent or not functional	565 (44.3)	228 (40.4)	337 (59.6)	1.1 (0.9–1.1)	0.427		
Present and functional	710 (55.7)	271 (38.2)	439 (61.8)				

AOR, Adjusted Odds Ratio; CI, Confidence Interval; COR, Crude Odds Ratio; n, number; STH, Soil-transmitted helminth; USD, United States Dollar.

Multivariate logistic regression of the above-mentioned statistically significant variables revealed that playing barefoot (AOR 1.6, 95% CI 1.1–2.2, and *p*-value 0.006), not washing hands after defecating and before eating (AOR 1.3, 95% CI 1.0–1.7, and *p*-value 0.043), having untrimmed nails (AOR 1.4, 95% CI 1.1–1.8, and *p*-value 0.010), and belonging to poor families (AOR 1.3, 95% CI 1.0–1.7, and *p*-value 0.038) as the risk factors associated with the predisposition of school children for getting STH (Table 4). Hookworm infection and other STHs (ascariasis and trichuriasis) are different in the aspect of life cycle of the parasites. Therefore, we did the analysis for risk factors between these two different infections, but there were no statistically significant difference between them.

## Discussion

In this cross-sectional study, we studied 1275 school children during eight-month period (May–December, 2022). The overall prevalence of STH among school children of Kandahar city was 39.1% with mean intensity of 97.8. Main risk factors associated with STH among school children were playing barefoot, not washing hands after defecating and before eating, having untrimmed nails, and belonging to poor families.

In our study, prevalence of STH among school children was 39.1%. This prevalence is more than the studies conducted in Cameroon (1%) [24], Nepal (3.1%) [25], Indonesia (10.1%) [26], China (14.1%) [27], Tajikistan (32%) [28], and Malaysia (37%) [29]. Contrary, prevalence in our study is less than reported in studies from Philippines (84.7%) [30], Ethiopia (84.4%) [31], Nigeria (83.3%) [32], and India (75.6%) [33]. The prevalence variances observed in different parts of the world (and even different areas of the same country) are multifactorial; including differences in stool examination techniques, geographical location, time of study, type of study, age of study participants, culture, socio-economic status, literacy levels/occupations of the parents or guardians, food consumption habits, personal hygiene behaviors, and playing habits and facilities of children in and outside the school [34]. Table 5 compares the prevalence of STH infections in school children among our study and two other studies conducted in Kandahar city in 2003 and 2017. In our study, the prevalence of STH is lower but prevalence of hookworm is higher than other two studies conducted in Kandahar. In 2017 study, no moderate or heavy intensity STH infections were observed. In 2003 study, moderate-severe intensity was observed only in *A*. *lumbricoides* infection. Decreased STH prevalence (39.1%) in our study can be contributed to the collection of only one stool sample instead of the standard three samples and Afghanistan ministry of public health (with the help of UN donor agencies) implementation of mass deworming interventions once in a year among school children throughout Afghanistan. Increased prevalence of hookworm in our study could be due to increased contact of school children to soil. In recent years, due to drought and decreased availability of ground water in Kandahar city, grass playgrounds have changed into soil playgrounds in nearly all schools.

**Table 5:** Prevalence of STH infections among school children in Kandahar city, in 2003, 2017, and 2022.

	2003 [19]	2017 [20]	2022 (This study)
Sample size (n)	257	452	1275
Any STH (%)	42.8	46.8	39.1
*Ascaris lumbricoides* (%)	37.4	45.5	29.4
Light intensity	91.7	100	90.4
Moderate intensity	8.3*	0	9.6
Heavy intensity		0	0
*Trichuris trichiura* (%)	7.8	1.4	12.1
Light intensity	100	100	97.4
Moderate intensity	0	0	2.6
Heavy intensity	0	0	0
Hookworm spp. (%)	0	0.5	8.7
Light intensity	0	100	95.5
Moderate intensity	0	0	4.5
Heavy intensity	0	0	0

* Moderate-heavy infection

STH, Soil-transmitted helminth.

The most prevalent STH in our study was *A*. *lumbricoides* (29.4%). The *A*. *lumbricoides* as the most common STH has also been reported in studies from Nepal (26.6%) [35], India (69.6%) [33], and Nigeria (75.6%) [36]. The reason behind the *A*. *lumbricoides* predominance could be associated to the long life of the female worm and has a fecundity rate of approximately 134,000–360,000 eggs per day for nearly 300 days. As a result, vast numbers of eggs are discharged into the human environment daily. Furthermore, the hard nature of these eggs to resist adverse environmental conditions more than other STHs can contribute to sustaining the transmission cycle for a longer period [37]. A study was conducted on 207 adults and 179 children visiting health facilities of Ghazni and Parwan provinces of Afghanistan. This study concluded that the most prevalent STH among children was *A*. *lumbricoides* (25.1% in Ghazni province and 10.8% in Parwan province) [16]. In 2003, a study conducted among school children of Kabul, Nangarhar, Farah, and Kandahar provinces of Afghanistan revealed that the most prevalent STH was *A*. *lumbricoides* with the prevalence of 41% (408/1001) in the school children of these four provinces of Afghanistan [19]. In 2017, another study conducted in Kabul, Balkh, Herat, Nangarhar and Kandahar provinces of Afghanistan showed that the most prevalent STH infection was the one with *A*. *lumbricoides* (25.7%) [20]. In 2020, a community-based study in rural children of Daman district in Kandahar Afghanistan also reported *A*. *lumbricoides* as the most prevalent STH species (18.7%) [21].

Aside from STH, the most prevalent (22.4%) intestinal parasite in our study was *G*. *intestinalis*. Similar results have been reported from another study in Afghanistan (14.5%) [16], Pakistan (28.9%) [38], Tajikistan (26.4%) [28], Kenya (26.1%) [39], and Peru (27.5) [40]. Evidence reveals that not only acute but chronic Giardiasis has an effect on the nutrition and health status of children [41]. Chronic intestinal protozoal infections are increasingly recognized as cause of undernutrition among children and have been proposed to be considered as neglected tropical diseases, causing morbidity in children comparable to infections caused by STHs [42].

Fortunately, severe intensity of STH infection was not observed in any child in this study. However, moderate intensity of *A*. *lumbricoides*, hookworm spp., and *T*. *trichiura* was present in 9.6%, 4.5%, and 2.6% of our study children, respectively. In study conducted during 2017 in Kandahar, no moderate-heavy intensity STH infections were observed [20]. This increase observed in our study could be contributed to the decrease in socio-economic status of people since the advent of Taliban in August 2021. Our result is higher than the WHO elimination target of STHs, which is defined as a <2% of moderate and heavy intensity due to STH infections [43]. Compared to our study, increased moderate-heavy intensity STH infections have been reported from Myanmar [44], Malaysia [45], Cameroon [46], and Rwanda [47]. Contrary, decreased moderate-heavy intensity STH infections have been reported from India [48], Ethiopia [37], and Nigeria [49].

Our study showed that children who were not washing hand after defecation and before eating were having statistically significant STH. Protective effects of handwashing have also been reported from Iran [50], Uzbekistan [51], China [27], India [52,53], Nepal [25], Lao [54], Indonesia [26], and Ethiopia [55]. In contrast, Wondemann and colleagues in Cuba reported a negative association between hand washing and infections [56]. This could be due to the lack of knowledge about transmission of the parasites or lack of awareness regarding health and hygiene habits among mothers [57].

In this study, living in poor families was a risk factor associated with increased STH infection among school children. Similar results have been reported in other studies [38,58,59]. It could be due to the reason that poor families have very little access to clean water, sanitation, and hygiene.

Our study revealed that walking barefoot is a risk factor for having increased STH infection. Similar results have been reported from Nepal [60], Thailand [61], Indonesia [26], Malawi [62], Ethiopia [63], and Kenya [64]. On the other hand, studies conducted in India did not notice any association between footwear usage and STH [65,66]. Walking barefoot is especially a risk factor for hookworms, as their larvae in the soil can penetrate into unbroken skin. Although walking barefoot is not directly related to infections of other helminths, but it indirectly leads to the infection when child touches the contaminated feet and eat with unwashed hands afterwards [67].

Having untrimmed nails was also a risk factor of increased STH infection in our study. This result is in accordance with researches reported from Ethiopia [68–70] and Thailand [71]. This could be due to the outdoor playing habits of school children on poor sanitation areas, which results in contamination of their hands. Dirt under fingernails may harbor different stages of parasites, which can be ingested during food eating and nail-biting or thumb sucking [69,71].

### Limitations

There were some limitations in our study. We obtained only one fecal sample instead of the ideal three consecutive samples due to unavailability of fund, the level of cooperation, and response of the caretakers and children. This might underestimate the real burden of STH. Also, this study did not focus on molecular assays and other techniques that are best to estimate the prevalence of STHs and differentiate different species of hookworm. We did not get data of clinical symptoms and underlying diseases of the children, which can be confounding factors for STH. Additional studies should be performed in different parts of the country.

## Conclusion

Based on the results of our study, more than one-third of the primary school children were infected with at least one STH species. This indicates that STHs are still a health problem among primary school children in Kandahar. Main risk factors associated with the predisposition of school children for getting STH were playing barefoot, children from poor families, children not washing hand after defecation/before eating, and children with untrimmed finger nails.

We recommend that improvement of socioeconomic status, sanitation, and health education to promote public awareness about health and hygiene. Periodic mass deworming programs are crucial for the control of STH infections in Afghanistan. Besides albendazole, praziquantel or niclosamide should be added to the deworming program, to cover *H*. *nana* and Taenia spp. too. Meanwhile, government and international donor agencies in Afghanistan should help in improving socio-economic status of the people through provision of basic facilities such as piped water, electricity, good housing, and proper toilets.

## Supporting information

S1 File Microsoft excel file with some of the data of this study. (XLSX)

## References

[1] World Health Organization. Soil-transmitted helminth infections. In: WHO. 2023 [cited 14 May 2023]. Available: https://www.who.int/news-room/fact-sheets/detail/soil-transmitted-helminth-infections

[2] Murphy E, Togbevi IC, Ibikounlé M, Avokpaho EFGA, Walson JL, Means AR. Soil-transmitted helminth surveillance in Benin: A mixed-methods analysis of factors influencing non-participation in longitudinal surveillance activities. PLoS Negl Trop Dis. 2023;17: e0010984. doi: 10.1371/JOURNAL.PNTD.0010984 36626399 PMC9831304

[3] Salam N, Azam S. Prevalence and distribution of soil-transmitted helminth infections in India. BMC Public Health. 2017;17: 201. doi: 10.1186/s12889-017-4113-2 28209148 PMC5311856

[4] World Health Organization. Preventive chemotherapy in human helminthiasis. Coordinated use of anthelminthic drugs in control interventions: a manual for health professionals and programme managers. Geneva: WHO; 2006.

[5] Parija S, Chidambaram M, Mandal J. Epidemiology and clinical features of soil-transmitted helminths. Trop Parasitol. 2017;7: 81–85. doi: 10.4103/tp.TP_27_17 29114484 PMC5652059

[6] Hailegebriel T, Nibret E, Munshea A. Prevalence of Soil-Transmitted Helminth Infection Among School-Aged Children of Ethiopia: A Systematic Review and Meta-Analysis. Infectious Diseases: Research and Treatment. 2020;13: 1–14. doi: 10.1177/1178633720962812 33088182 PMC7543112

[7] Jourdan PM, Lamberton PHL, Fenwick A, Addiss DG. Soil-transmitted helminth infections. Lancet. 2018;391: 252–265. doi: 10.1016/S0140-6736(17)31930-X 28882382

[8] Awasthi S, Bundy DAP, Savioli L. Helminthic infections. BMJ; 2003; 327(7412):431–433. doi: 10.1136/bmj.327.7412.431 12933732 PMC188497

[9] Crompton DWT. Ascariasis and childhood malnutrition. Trans R Soc Trop Med Hyg. 1992;86: 577–579. doi: 10.1016/0035-9203(92)90133-w 1287902

[10] Crompton DWT, Nesheim MC. Nutritional impact of intestinal helminthiasis during the human life cycle. Annu Rev Nutr. 2002;22:35–59. doi: 10.1146/annurev.nutr.22.120501.134539 12055337

[11] Oberhelman RA, Guerrero ES, Fernandez ML, Silio M, Mercado D, Comiskey N, et al. Correlations between intestinal parasitosis, physical growth, and psychomotor development among infants and children from rural Nicaragua. American Journal of Tropical Medicine and Hygiene. 1998;58: 470–475. doi: 10.4269/ajtmh.1998.58.470 9574794

[12] Girum T, Wasie A. The Effect of Deworming School Children on Anemia Prevalence: A Systematic Review and Meta-Analysis. Open Nurs J. 2018;12: 155–161. doi: 10.2174/1874434601812010155 30197721 PMC6110060

[13] Opoku EC, Olsen A, Browne E, Hodgson A, Awoonor-Williams JK, Yelifari L, et al. Impact of combined intermittent preventive treatment of malaria and helminths on anaemia, sustained attention, and recall in Northern Ghanaian schoolchildren. Glob Health Action. 2016;9: 32197. doi: 10.3402/gha.v9.32197 27633035 PMC5025525

[14] Mireku MO, Davidson LL, Koura GK, Ouédraogo S, Boivin MJ, Xiong X, et al. Prenatal hemoglobin levels and early cognitive and motor functions of one-year-old children. Pediatrics. 2015;136: e76–e83. doi: 10.1542/peds.2015-0491 26055847 PMC9924076

[15] Wagner AL, Mubarak MY, Johnson LE, Porth JM, Yousif JE, Boulton ML. Trends of vaccine-preventable diseases in Afghanistan from the Disease Early Warning System, 2009–2015. PLoS One. 2017;12: e0178677. doi: 10.1371/journal.pone.0178677 28570694 PMC5453561

[16] Korzeniewski K, Chung WC, Augustynowicz A, Lass A, Ik KJ. Current status of intestinal parasitic infections among inhabitants of the Ghazni and Parwan Provinces, Afghanistan. Family Medicine and Primary Care Review. 2017;19: 23–28. doi: 10.5114/fmpcr.2017.65087

[17] Elyan DS, Monestersky JH, Wasfy MO, Noormal B, Oyofo BA. Capacity building of public health laboratories in Afghanistan: challenges and successes (2007–2011). Eastern Mediterranean Health Journal. 2014;20: 112–119. 24945560

[18] Wallace MR, Hale BR, Utz GC, Olson PE, Earhart KC, Thornton SA, et al. Endemic infectious diseases of Afghanistan. Clinical Infectious Diseases. 2002;34: S171–S207. doi: 10.1086/340704 12019465

[19] Ramsan M, Gabrielli A-F, Tsogzolmaa D, Bojang B, Naumann C, Khoshal MH, et al. Distribution, prevalence and intensity of Soil-transmitted helminth infections among Afghan schoolchildren, 2003. J Helminthol. 2005;79: 381–384. Available: /pmc/articles/PMC5630093/16336723 10.1079/joh2005316 PMC5630093

[20] Safi N, Warusavithana S, Shah Alawi SA, Atta H, Montresor A, Gabrielli AF. Elimination of morbidity due to soil-transmitted helminthiases among Afghan schoolchildren. Acta Trop. 2019;197: 105035. doi: 10.1016/j.actatropica.2019.05.026 31128094 PMC7612416

[21] Rahimi BA, Mahboobi BA, Wafa MH, Sahrai MS, Stanikzai MH, Taylor WR. Prevalence and associated risk factors of soil-transmitted helminth infections in Kandahar, Afghanistan. BMC Infect Dis. 2022;22: 361. doi: 10.1186/s12879-022-07336-z 35410154 PMC9003950

[22] Kandahar Directorate of Education. Yearly Report of Kandahar Province Directorate of Education. In: Afghanistan Ministry of Education. 2021 [cited 10 Aug 2023]. Available: https://moe.gov.af/sites/default/files/2021-02/%DA%AF%D8%B2%D8%A7%D8%B1%D8%B4%20%D9%88%D9%84%D8%A7%DB%8C%D8%AA%DB%8C%20%DA%A9%D9%86%D8%AF%D9%87%D8%A7%D8%B1.pdf

[23] WHO Expert Committee on the Control of Schistosomiasis & World Health Organization. Prevention and control of schistosomiasis and soil-transmitted helminthiasis: report of a WHO expert committee. In: World Health Organization. 2002 [cited 28 Jan 2023]. Available: https://apps.who.int/iris/handle/10665/42588 12592987

[24] Tabi ESB, Eyong EM, Akum EA, Löve J, Cumber SN. Soil-transmitted Helminth infection in the Tiko Health District, South West Region of Cameroon: a post-intervention survey on prevalence and intensity of infection among primary school children. Pan Afr Med J. 2018;30: 74. doi: 10.11604/pamj.2018.30.74.15676 30344858 PMC6191252

[25] Tandukar S, Ansari S, Adhikari N, Shrestha A, Gautam J, Sharma B, et al. Intestinal parasitosis in school children of Lalitpur district of Nepal. BMC Res Notes. 2013;6: 449. doi: 10.1186/1756-0500-6-449 24207086 PMC3829703

[26] Arta Suryantari SA. Prevalence, intensity and risk factors of soil transmitted helminths infections among elementary school students in Ngis village, Karangasem district, Bali. Indonesian Journal of Tropical and Infectious Disease. 2019;7: 137. doi: 10.20473/IJTID.V7I6.9952

[27] Balen J, Raso G, Li YS, Zhao ZY, Yuan LP, Williams GM, et al. Risk factors for helminth infections in a rural and a peri-urban setting of the Dongting Lake area, People’s Republic of China. Int J Parasitol. 2011;41: 1165–1173. doi: 10.1016/j.ijpara.2011.07.006 21854780

[28] Matthys B, Bobieva M, Karimova G, Mengliboeva Z, Jean-Richard V, Hoimnazarova M, et al. Prevalence and risk factors of helminths and intestinal protozoa infections among children from primary schools in western Tajikistan. Parasit Vectors. 2011;4: 1–13. doi: 10.1186/1756-3305-4-195 21981979 PMC3205355

[29] Huat LB, Mitra AK, Jamil NIN, Dam PC, Mohamed HJJ, Muda WAMW. Prevalence and Risk Factors of Intestinal Helminth Infection Among Rural Malay Children. J Glob Infect Dis. 2012;4: 10. doi: 10.4103/0974-777X.93753 22529621 PMC3326951

[30] Papier K, Williams GM, Luceres-Catubig R, Ahmed F, Olveda RM, McManus DP, et al. Childhood Malnutrition and Parasitic Helminth Interactions. Clinical Infectious Diseases. 2014;59: 234–243. doi: 10.1093/cid/ciu211 24704723

[31] Yeshanew S, Bekana T, Truneh Z, Tadege M, Abich E, Dessie H. Soil-transmitted helminthiasis and undernutrition among schoolchildren in Mettu town, Southwest Ethiopia. Sci Rep. 2022;12: 3614. doi: 10.1038/s41598-022-07669-4 35256678 PMC8901616

[32] Ibidapo CA, Okwa O. The prevalence and intensity of soil transmitted helminths in a rural community, Lagos suburb, South West Nigeria. Int J Agric Biol. 2008;10: 89–92.

[33] Ganguly S, Barkataki S, Karmakar S, Sanga P, Boopathi K, Kanagasabai K, et al. High prevalence of soil-transmitted helminth infections among primary school children, Uttar Pradesh, India, 2015. Infect Dis Poverty. 2017;6: 139. doi: 10.1186/s40249-017-0354-7 28988538 PMC5632835

[34] Belyhun Y, Medhin G, Amberbir A, Erko B, Hanlon C, Alem A, et al. Prevalence and risk factors for soil-transmitted helminth infection in mothers and their infants in Butajira, Ethiopia: A population based study. BMC Public Health. 2010;10: 1–7. doi: 10.1186/1471-2458-10-21/TABLES/4 20085635 PMC2835680

[35] Khadka KS, Kaphle HP, Gurung K, Shah Y, Sigdel M. Study of Intestinal Parasitosis among School Going Children in Pokhara, Nepal. Journal of Health and Allied Sciences. 2013;3: 47–50. doi: 10.37107/JHAS.54

[36] Osazuwa Ayo OM, Imade P. A significant association between intestinal helminth infection and anaemia burden in children in rural communities of Edo state, Nigeria. N Am J Med Sci. 2011;3: 30–34. doi: 10.4297/najms.2011.330 22540060 PMC3336930

[37] Eyayu T, Yimer G, Workineh L, Tiruneh T, Sema M, Legese B, et al. Prevalence, intensity of infection and associated risk factors of soil-transmitted helminth infections among school children at Tachgayint woreda, Northcentral Ethiopia. PLoS One. 2022;17: e0266333. doi: 10.1371/journal.pone.0266333 35395035 PMC8993015

[38] Mehraj V, Hatcher J, Akhtar S, Rafique G, Beg MA. Prevalence and Factors Associated with Intestinal Parasitic Infection among Children in an Urban Slum of Karachi. PLoS One. 2008;3: e3680. doi: 10.1371/journal.pone.0003680 18997865 PMC2577067

[39] Obala AA, Simiyu CJ, Odhiambo DO, Nanyu V, Chege P, Downing R, et al. Webuye Health and Demographic Surveillance Systems Baseline Survey of Soil-Transmitted Helminths and Intestinal Protozoa among Children up to Five Years. J Trop Med. 2013;2013: 734562. doi: 10.1155/2013/734562 23533444 PMC3600298

[40] Cabada M, Goodrich M, Graham B, Villanueva-Meyer P, Deichsel E, Lopez M, et al. Prevalence of intestinal helminths, anemia, and malnutrition in Paucartambo, Peru. Rev Panam Salud Publica. 2015;37: 69–75. 25915010

[41] Gutiérrez EJ, Pineda V, Calzada JE, Guerrant RL, Neto JBL, Pinkerton RC, et al. Enteric parasites and enteroaggregative Escherichia coli in children from Cañazas County, Veraguas Province, Panama. Am J Trop Med Hyg. 2014;91: 267–272. doi: 10.4269/AJTMH.13-0438 24980494 PMC4125247

[42] Bartelt LA, Lima AAM, Kosek M, Peñataro Yori P, Lee G, Guerrant RL. “Barriers" to Child Development and Human Potential: The Case for Including the “Neglected Enteric Protozoa" (NEP) and Other Enteropathy-Associated Pathogens in the NTDs. PLoS Negl Trop Dis. 2013;7: e2125. doi: 10.1371/journal.pntd.0002125 23593514 PMC3623703

[43] World Health Organization. Ending the neglect to attain the Sustainable Development Goals: A road map for neglected tropical diseases 2021–2030. In: WHO. 2021 [cited 13 Aug 2023]. Available: https://www.who.int/publications/i/item/9789240010352

[44] Aung E, Han KT, Gordon CA, Hlaing NN, Aye MM, Htun MW, et al. High prevalence of soil-transmitted helminth infections in Myanmar schoolchildren. Infect Dis Poverty. 2022;11: 28. doi: 10.1186/s40249-022-00952-6 35272701 PMC8908594

[45] Muslim A, Sofian SM, Shaari SA, Hoh BP, Lim YAL. Prevalence, intensity and associated risk factors of soil transmitted helminth infections: A comparison between Negritos (indigenous) in inland jungle and those in resettlement at town peripheries. PLoS Negl Trop Dis. 2019;13: e0007331. doi: 10.1371/journal.pntd.0007331 31009476 PMC6497322

[46] Ruth MMR, Cedric Y, Malla ME, Nadia NAC, Aime TN, Leonelle M, et al. Intestinal Helminth Infections and Associated Risk Factors among School-Aged Children of Bamendjou Community, West Region of Cameroon. J Parasitol Res. 2021;2021. doi: 10.1155/2021/6665586 33981454 PMC8088379

[47] Kabatende J, Barry A, Mugisha M, Ntirenganya L, Bergman U, Bienvenu E, et al. Efficacy of Single-Dose Albendazole for the Treatment of Soil-Transmitted Helminthic Infections among School Children in Rwanda—A Prospective Cohort Study. Pharmaceuticals. 2023;16: 139. doi: 10.3390/ph16020139 37259291 PMC9964298

[48] Ganguly S, Barkataki S, Sanga P, Boopathi K, Kanagasabai K, Devika S, et al. Epidemiology of Soil-Transmitted Helminth Infections among Primary School Children in the States of Chhattisgarh, Telangana, and Tripura, India, 2015–2016. Am J Trop Med Hyg. 2022;107: 122–129. doi: 10.4269/ajtmh.21-1185 35576946 PMC9294677

[49] Griswold E, Eigege A, Adelamo S, Mancha B, Kenrick N, Sambo Y, et al. Impact of Three to Five Rounds of Mass Drug Administration on Schistosomiasis and Soil-Transmitted Helminths in School-Aged Children in North-Central Nigeria. Am J Trop Med Hyg. 2022;107: 132–142. doi: 10.4269/ajtmh.21-1207 35576949 PMC9294711

[50] Daryani A, Sharif M, Nasrolahei M, Khalilian A, Mohammadi A, Barzegar G. Epidemiological survey of the prevalence of intestinal parasites among schoolchildren in Sari, northern Iran. Trans R Soc Trop Med Hyg. 2012;106: 455–459. doi: 10.1016/j.trstmh.2012.05.010 22703897

[51] Gungoren B, Latipov R, Regallet G, Musabaev E. Effect of hygiene promotion on the risk of reinfection rate of intestinal parasites in children in rural Uzbekistan. Trans R Soc Trop Med Hyg. 2007;101: 564–569. doi: 10.1016/j.trstmh.2007.02.011 17418321

[52] Kaliappan SP, George S, Francis MR, Kattula D, Sarkar R, Minz S, et al. Prevalence and clustering of soil-transmitted helminth infections in a tribal area in southern India. Tropical Medicine & International Health. 2013;18: 1452–1462. doi: 10.1111/TMI.12205 24237860 PMC3923153

[53] Awasthi S, Verma T, Kotecha P, Venkatesh V, Joshi V, Roy S. Prevalence and risk factors associated with worm infestation in pre-school children (6–23 months) in selected blocks of Uttar Pradesh and Jharkhand, India. Indian J Med Sci. 2008;62: 484–491. doi: 10.4103/0019-5359.48552 19265242

[54] Hohmann H, Panzer S, Phimpachan C, Southivong C, Schelp F. Relationship of intestinal parasites to the environment and to behavioral factors in children in the Bolikhamxay Province of Lao PDR. Southeast Asian J Trop Med Public Health. 2001;32: 4–13. 11485093

[55] Nasr NA, Al-Mekhlafi HM, Ahmed A, Roslan MA, Bulgiba A. Towards an effective control programme of soil-transmitted helminth infections among Orang Asli in rural Malaysia. Part 1: Prevalence and associated key factors. Parasit Vectors. 2013;6: 1–12. doi: 10.1186/1756-3305-6-27 23356952 PMC3564908

[56] Wördemann M, Polman K, Menocal Heredia LT, Junco Diaz R, Collado Madurga AM, Núñez Fernández FA, et al. Prevalence and risk factors of intestinal parasites in Cuban children. Tropical medicine & international health. 2006;11: 1813–1820. doi: 10.1111/j.1365-3156.2006.01745.x 17176346

[57] Messaad SA, Laboudi M, Moumni M, Sarhane B, Belghyti D, Kharrim KE. Children Intestinal parasites related to socio-economic factors in Salé Hospital, Morocco. Int J Innov Appl Stud. 2014;8: 833–840.

[58] Quihui L, Valencia ME, Crompton DWT, Phillips S, Hagan P, Morales G, et al. Role of the employment status and education of mothers in the prevalence of intestinal parasitic infections in Mexican rural schoolchildren. BMC Public Health. 2006;6: 225. doi: 10.1186/1471-2458-6-225 16956417 PMC1584408

[59] Nematian J, Nematian E, Gholamrezanezhad A, Asgari AA. Prevalence of intestinal parasitic infections and their relation with socio-economic factors and hygienic habits in Tehran primary school students. Acta Trop. 2004;92: 179–186. doi: 10.1016/j.actatropica.2004.06.010 15533285

[60] Parajuli RP, Umezaki M, Watanabe C. Behavioral and nutritional factors and geohelminth infection among two ethnic groups in the Terai region, Nepal. American Journal of Human Biology. 2009;21: 98–104. doi: 10.1002/ajhb.20825 18802944

[61] Jiraanankul V, Aphijirawat W, Mungthin M, Khositnithikul R, Rangsin R, Traub RJ, et al. Incidence and risk factors of hookworm infection in a rural community of central Thailand. American Journal of Tropical Medicine and Hygiene. 2011;84: 594–598. doi: 10.4269/ajtmh.2011.10-0189 21460016 PMC3062455

[62] Phiri K, Whitty CJ, Graham S, Ssembatya-Lule G. Urban/rural differences in prevalence and risk factors for intestinal helminth infection in southern Malawi. Ann Trop Med Parasitol. 2000;94: 381–387. doi: 10.1080/00034983.2000.11813553 10945048

[63] Erosie L, Merid Y, Ashiko A, Ayine M, Balihu A, Muzeyin S, et al. Prevalence of Hookworm infection and haemoglobin status among rural elementary school children in Southern Ethiopia. Ethiopian Journal of Health Development. 2002;16: 113–115. doi: 10.4314/EJHD.V16I1.9833

[64] Freeman MC, Chard AN, Nikolay B, Garn J V., Okoyo C, Kihara J, et al. Associations between school- and household-level water, sanitation and hygiene conditions and soil-transmitted helminth infection among Kenyan school children. Parasit Vectors. 2015;8: 412. doi: 10.1186/s13071-015-1024-x 26248869 PMC4528701

[65] Kattula D, Sarkar R, Ajjampur S, Minz S, Levecke B, Muliyil J, et al. Prevalence & risk factors for soil transmitted helminth infection among school children in south India. Indian J Med Res. 2014;139: 76–82. 24604041 PMC3994744

[66] Puhalenthi K, Chandrababu C, Maniprabhu S, Vijayakumar K, Thomas M, Mini S. Prevalence of Geohelminthic Infection and Its Risk Factors Among School Children in Thiruvananthapuram, Kerala, India. Cureus. 2023;15: e34116. doi: 10.7759/cureus.34116 36843715 PMC9946816

[67] Anunobi JT, Okoye IC, Aguzie IO, Ndukwe YE, Okpasuo OJ. Risk of soil-transmitted helminthiasis among agrarian communities of Kogi state, Nigeria. Ann Glob Health. 2019;85: 120, 1–13. doi: 10.5334/aogh.2569 31517465 PMC6743035

[68] Ibrahim T, Zemene E, Asres Y, Seyoum D, Tiruneh A, Gedefaw L, et al. Epidemiology of soil-transmitted helminths and Schistosoma mansoni: a base-line survey among school children, Ejaji, Ethiopia. J Infect Dev Ctries. 2018;12: 1134–1141. doi: 10.3855/jidc.9665 32027616

[69] Eyamo T, Girma M, Alemayehu T, Bedewi Z. Soil-Transmitted Helminths and Other Intestinal Parasites Among Schoolchildren in Southern Ethiopia. Res Rep Trop Med. 2019;10: 137–143. doi: 10.2147/RRTM.S210200 31695554 PMC6817342

[70] Samuel F, Demsew A, Alem Y, Hailesilassie Y. Soil transmitted Helminthiasis and associated risk factors among elementary school children in Ambo town, western Ethiopia. BMC Public Health. 2017;17: 1–7. doi: 10.1186/S12889-017-4809-3 29017470 PMC5634961

[71] Laoraksawong P, Suntaraluk A, Kongnil W, Pongpanitanont P, Janwan P. Prevalence of Soil–Transmitted Helminth Infections and Associated Risk Factors among Schoolchildren in Nakhon Si Thammarat, Thailand. Iran J Parasitol. 2020;15: 440–445. doi: 10.18502/ijpa.v15i3.4210 33082810 PMC7548470

