# Supplementary material for: Prevalence of soil-transmitted helminths and associated risk factors among primary school children in Kandahar, Afghanistan: A cross-sectional analytical study
Source: PLoS Negl Trop Dis. 2023 Sep 11;17(9):e0011614. doi: 10.1371/journal.pntd.0011614 (PMC10513306; doi:10.1371/journal.pntd.0011614)
Supplement: Attachment — Submitted filename: Responses to Reviewer Comments.docx [file pntd.0011614.s002.docx]

Dear reviewers,

Many thanks for your useful and important comments.

Our team went through all the comments and brought changes in the manuscript based on your comments.

Following are your comments, followed by our answers:

**Reviewer's Responses to Questions**

Key Review Criteria Required for Acceptance?

As you describe the new analyses required for acceptance, please consider the following:

- -Are the objectives of the study clearly articulated with a clear testable hypothesis stated?
- -Is the study design appropriate to address the stated objectives?
- -Is the population clearly described and appropriate for the hypothesis being tested?
- -Is the sample size sufficient to ensure adequate power to address the hypothesis being tested?
- -Were correct statistical analysis used to support conclusions?
- -Are there concerns about ethical or regulatory requirements being met?

Reviewer #1: LAB TECHNIQUES

It is not clear what lab technique was applied to detect which parasite.

Was the saline wet mount method used for protozoa and the Kato-Katz for helminths?

**Answer**: Thanks. Great point. Now I have made it clear in the manuscript and mentioned.

Yes, saline wet mount method was used for protozoa and the Kato-Katz for intestinal helminths.

SCHOOL ENROLMENT

Please clarify the primary school enrolment rate (%) in Kandahar, and comment on whether

children included in the study can be considered as representative the school-age population

of the city.

**Answer**: Thanks for the comment. Now the following information is added in the Methods part of manuscript:

Based on the unpublished data, the total population of Kandahar city is 860,000 with primary school age population of 121,000. During 2022, there were a total of 17,950 (14.8% of primary school age population) primary school children with 11844 (66%) boys and 6,106 (34.0%) girls. So, our sample size constitutes 7.1% of school population while 1.1% of all primary school-age children of Kandahar city.

Primary school enrolment rate is nearly 14.8% in Kandahar city.

Reviewer #2: Yes, but some information is missing. Please refer to general comments section.

**Results**

-Does the analysis presented match the analysis plan?

-Are the results clearly and completely presented?

-Are the figures (Tables, Images) of sufficient quality for clarity?

Reviewer #1: Please clarify what % of the entire schoolchildren population of Kandahar (or
what % of schools) were included in the survey (to understand how representative the sample was).

**Answer**: OK. Thanks. Now the following information has been added to in the Methods part of manuscript:

Based on the unpublished data, the total population of Kandahar city is 860,000 with primary school age population of 121,000. During 2022, there were a total of 17,950 (14.8% of primary school age population) primary school children with 11844 (66%) boys and 6,106 (34.0%) girls. So, our sample size constitutes 7.1% of school population while 1.1% of all primary school-age children of Kandahar city.

Reviewer #2: Yes, but some needs to be revised. Please refer to general comments section.

--------------------

**Conclusions**

-Are the conclusions supported by the data presented?

-Are the limitations of analysis clearly described?

-Do the authors discuss how these data can be helpful to advance our understanding of the

topic under study?

-Is public health relevance addressed?

Reviewer #1: These comments cover both sections "Discussion" and "Conclusions"

(1) Please add comments on intensity of STH infections and its implications on morbidity.

**Answer**: OK. Now comments on intensity of STH infections and its implication on morbidity has been added.

(2) Please add information on intensity of STH infections to Table 5 and draw a comparison: it

looks like that in 2017 no moderate-intensity infections were found but in your survey you do

find some moderate-intensity infections.

**Answer**: Thanks. For comparison, now more information on STH intensity of 2003 and 2017 studies have been added to Table 5. Also, this comparison has been discussed in “Discussion” part of the manuscript.

(3) Please add details on the current status of STH deworming activities carried out in

Afghanistan in general and in Kandahar more specifically.

**Answer**: Now (as in previous years), STH deworming is carried out by ministry of public health with the help of UN agencies (WHO and UNICEF), once a year for all school children in all provinces of Afghanistan (including Kandahar).

(4) Although the title of the paper implies that its focus is on STH, data on intestinal protozoa

and on other helminths were nevertheless collected and presented. It would be useful to

include these in the discussion and draw a comparison with information taken from previously implemented surveys.

**Answer**: Thanks. OK. Now data of intestinal protozoa has been added and discussed in the discussion section of the manuscript.

(5) The "Discussion" section contains some repetition of information already presented in the

"Introduction" - please streamline as necessary

**Answer**: OK. Now the repeated information has been streamlined. Now the repeated information has been removed from introduction section.

Reviewer #2: Yes

--------------------

**Editorial and Data Presentation Modifications?**

Use this section for editorial suggestions as well as relatively minor modifications of existing data that would enhance clarity. If the only modifications needed are minor and/or editorial, you may wish to recommend “Minor Revision” or “Accept”.

Reviewer #1: Please refer to above comments

Reviewer #2: (No Response)

--------------------

**Summary and General Comments**

Use this section to provide overall comments, discuss strengths/weaknesses of the study, novelty, significance, general execution and scholarship. You may also include additional comments for the author, including concerns about dual publication, research ethics, or publication ethics. If requesting major revision, please articulate the new experiments that are needed.

Reviewer #1: A descriptive study contributing to the body of evidence on STH in Afghanistan.

Some limitations:

(1) The survey is focused on a single province, Kandahar. It looks like there is some overlapping with a paper published by the same first author in 2022 (with very similar title), although this was focused on a single district; in light of this, sentences such as "The risk factors of STH among school children are unknown in Afghanistan" (Background) do not seem to be appropriate.

**Answer**: Thanks for the comment. Yes, in 2022 I have published an article, but that research is different from this one. Following are the main differences between these two researches:

|  | **Article published in 2022** | **This study** |
| --- | --- | --- |
| Study area | Daman district (rural area) of Kandahar province | Kandahar city (urban area) |
| Type of research | Community-based | School-based |
| Study population | Community children <15 years old | Primary school children 6-12 years old |
| Data collection period | 5 months (June–October, 2020) | 8 months (May–December, 2022) |
| Diagnosis method | Only Saline wet mount microscopy | Saline wet mount microscopy + Kato-Katz  technique |

OK. Now the sentence "The risk factors of STH among school children are unknown in Afghanistan" is removed from the Abstract and Background.

(3) Discussion on STH should focus more on intensity of infection (see comments above)

**Answer**: OK. Thanks for the comment. Now I have discussed the intensity of infection in the discussion section.

Reviewer #2: The authors demonstrated the prevalence of STH and associated risk factors among PSAC in Afghanistan. Although there is limitation in analyzing one sample per individual, it can provide evidence for high prevalence and associated risk factors that need interventions. This article has strength in including large number of PSACs and investing risk factors in the same individuals, but more detailed descriptions and analysis are needed.

These are some inquiry and suggestions.

1. Authors stated that children who received any anti-helminthic treatment in the previous three months were excluded, but the number excluded by this criteria was not described. On page 9, only those whose parents refused or who failed to submit their fecal samples were described. And it is hard to know previous treatment history or presence of chronic disease before the investigation. Please describe how it was investigated and how many were excluded by this criteria. If the previous treatment was based on the stool exam or symptom based, this could be another reason why this study shows lower prevalence resulting from selection bias.

**Answer**: Thanks for the comment. Yes, we had children with previous anti-helminthic treatment history or history of having chronic diseases. They were very small in number (i.e., 6 with previous anti-helminthic treatment history and 2 had history of chronic diseases) therefore we mistakenly ignored them and put them in the category of children who failed to submit their fecal samples. Now this is corrected and all the numbers have been put in the “methods” section of the manuscript.

2. (Table 1) The percentage next to the should be described the other way, describing proportions within the boys or girls. The current way of description is affected by the total number of boys and girls included, and cannot be used to describe differences in proportions between boys and girls.

**Answer**: OK. Thanks. Now the percentages have been changed in Table 1 based on your comment.

3. Hookworm infection and other STHs (ascariasis and trichuriasis) are different in the aspect of life cycle of the parasites. If there was difference in the result of risk factor analysis between these two different infections, please describe it. Even if not, please state it in the manuscript. For instance, it would be interesting to see if barefoot is also associated with ascariasis or trichuriasis in this study.

**Answer**: OK. Thanks for the great point. We did the analysis for risk factors between these two different infections, but there was no statistically significant difference between them. Now this is mentioned in the Results section of the manuscript.

4. It seems this study include a number of families with more than 5 people. Can authors identify household information? If many of the individuals are coming from the same household, the statistics can be over represented in the household with many PSACs. Please indicate if authors have household information, and if there are substantial number, it would be beneficial to provide analysis with household data.

**Answer**: Thanks. This is great point. Our team was aware of this issue before. If more than one children are coming from the same household, the statistics can be over represented in the household with many primary school age children. To avoid this issue, only one child per house was enrolled in this study. We had not mentioned this in our manuscript. Now it is mentioned in the Methods part of the manuscript. Thanks once again for the reminder.

Minor inquiry and suggestions.

It seems that children were questioned, not guardians were according to the description in methods. Please indicate if the guardians were questioned.

**Answer**: Thanks for the comment. We interviewed both children and their parents/care-takers. Now it is clearly mentioned in the Methods section.

___________________________________________________________________________________

PLOS authors have the option to publish the peer review history of their article (<ahref="https://journals.plos.org/plosntds/s/editorial-and-peer-review-process#loc-peer-reviewhistory" target="_blank">what does this mean?</a>). If published, this will include your full peer

review and any attached files.

If you choose “no”, your identity will remain anonymous but your review may still be made

public.

Do you want your identity to be public for this peer review? For information about this

choice, including consent withdrawal, please see our <a href="https://www.plos.org/privacypolicy" target="_blank">Privacy Policy</a>.

Reviewer #1: No

Reviewer #2: Yes: Hyun Beom Song

Figure Files:

While revising your submission, please upload your figure files to the Preflight Analysis and
Conversion Engine (PACE) digital diagnostic tool, https://pacev2.apexcovantage.com. PACE
helps ensure that figures meet PLOS requirements. To use PACE, you must first register as a

user. Then, login and navigate to the UPLOAD tab, where you will find detailed instructions on

how to use the tool. If you encounter any issues or have any questions when using PACE,

please email us at figures@plos.org.

Data Requirements:

Please note that, as a condition of publication, PLOS' data policy requires that you make
available all data used to draw the conclusions outlined in your manuscript. Data must be
deposited in an appropriate repository, included within the body of the manuscript, or uploaded

as supporting information. This includes all numerical values that were used to generate

graphs, histograms etc.. For an example see here:

http://www.plosbiology.org/article/info%3Adoi%2F10.1371%2Fjournal.pbio.1001908#s5.

Reproducibility:

To enhance the reproducibility of your results, we recommend that you deposit your laboratory
protocols in protocols.io, where a protocol can be assigned its own identifier (DOI) such that it
can be cited independently in the future. Additionally, PLOS ONE offers an option to publish

peer-reviewed clinical study protocols. Read more information on sharing protocols at

https://plos.org/protocols?utm_medium=editorialemail&utm_source=authorletters&utm_campaign=protocols

References

Please review your reference list to ensure that it is complete and correct. If you have cited
papers that have been retracted, please include the rationale for doing so in the manuscript
text, or remove these references and replace them with relevant current references. Any
changes to the reference list should be mentioned in the rebuttal letter that accompanies your
revised manuscript. If you need to cite a retracted article, indicate the article's retracted status
in the References list and also include a citation and full reference for the retraction notice.

__________________________________________________

In compliance with data protection regulations, you may request that we remove your personal
registration details at any time. (Use the following URL:
https://www.editorialmanager.com/pntd/login.asp?a=r). Please contact the publication office if
you have any questions.
